# Molecular Weight-Dependent Physical and Photovoltaic Properties of Poly(3-alkylthiophene)s with Butyl, Hexyl, and Octyl Side-Chains

**DOI:** 10.3390/polym13193440

**Published:** 2021-10-07

**Authors:** Thanh-Danh Nguyen, Van-Hai Nguyen, Jongwoo Song, Jongdeok An, Ngoc-Thuan Truong, Chi-Hien Dang, Chan Im

**Affiliations:** 1 Department of Chemistry, Konkuk University, 120 Neungdong-ro, Gwangjin-gu, Seoul 05029, Korea; ntdanh@ict.vast.vn (T.-D.N.); vanhai0203@gmail.com (V.-H.N.); birdjw@konkuk.ac.kr (J.S.); anhijuk@konkuk.ac.kr (J.A.); ngocthuan1810@gmail.com (N.-T.T.); 2 Institute of Chemical Technology, Vietnam Academy of Science and Technology, 1A TL29 Street, Thanh Loc Ward, District 5, Ho Chi Minh City 70000, Vietnam; dangchihien@gmail.com

**Keywords:** GRIM polymerization, polythiophene, molecular weight, side-chain length, organic photovoltaics

## Abstract

A series of poly-3-alkylthiophenes (P3ATs) with butyl (P3BT), hexyl (P3HT), and octyl (P3OT) side-chains and well-defined molecular weights (MWs) were synthesized using Grignard metathesis polymerization. The MWs of P3HTs and P3OTs obtained via gel permeation chromatography agreed well with the calculated MWs ranging from approximately 10 to 70 kDa. Differential scanning calorimetry results showed that the crystalline melting temperature increased with increasing MWs and decreasing alkyl side-chain length, whereas the crystallinity of the P3ATs increased with the growth of MWs. An MW-dependent red shift was observed in the UV–Vis and photoluminiscence spectra of the P3ATs in solution, which might be a strong evidence for the extended effective conjugation occurring in polymers with longer chain lengths. The photoluminescence quantum yields of pristine films in all polymers were lower than those of the diluted solutions, whereas they were higher than those of the phenyl-C_61_-butyric acid methyl ester-blended films. The UV–Vis spectra of the films showed fine structures with pronounced red shifts, and the interchain interaction-induced features were weakly dependent on the MW but significantly dependent on the alkyl side-chain length. The photovoltaic device performances of the P3BT and P3HT samples significantly improved upon blending with a fullerene derivative and subsequent annealing, whereas those of P3OTs mostly degraded, particularly after annealing. The optimal power conversion efficiencies of P3BT, P3HT, and P3OT were 2.4%, 3.6%, and 1.5%, respectively, after annealing with MWs of ~11, ~39, and ~38 kDa, respectively.

## 1. Introduction

Poly-3-alkylthiophenes (P3ATs) have been intensively investigated and developed for use in various organic electronic devices, such as organic light-emitting diodes [1], organic photovoltaic cells (OPVs) [2,3,4,5,6], and organic field-effect transistors [7,8], because of their easy deposition from solution onto various types of substrates and low preparation cost [9]. In OPVs, the polymer is known to be an effective semiconducting material with a relatively high hole mobility [10,11,12] and self-assembly capacity [13,14].

P3ATs with simple chemical structures can be controllably synthesized using numerous methods, including McCullough [15,16,17], Rieke [18,19,20], and Grignard metathesis polymerization (GRIM) [21,22,23] syntheses. Among these, the GRIM method developed by Yokoyawa et al. [24,25] with a high regioregularity (RR) of 98% head-to-tail coupling is currently preferred. This method is based on nickel-catalyzed cross-coupling polymerization and proceeds via living chain growth according to a catalyst transfer mechanism; it is attractive because of the ready availability of required reagents and relatively mild reaction conditions, as shown in Scheme S1 [25]. Two equivalents of Grignard monomer are coupled using as catalyst Ni(dppp)Cl_2_. The Ni species intramolecularly transfers to the terminal C–Br bond by walking through the π-conjugation to afford tail-to-tail dimers. The dimers then act as virtual initiators for the polymerization of the Grignard monomer via transmetalation. When the initiation rate is faster than or similar to the propagation rate, the molecular weight (MW) of the resulting polymer can be controlled through the feed ratio [monomer]_0_/[Ni]_0_ [26,27,28].

Regioregular P3AT (rr P3AT) tends to adopt a layered structure, wherein laterally packed thiophene main chains are separated by alkyl side-chains. The RR of P3AT can affect the charge transport and absorption in thin films, leading to enhanced performance in photovoltaic devices. The general consensus is that OPV devices can attain high efficiencies when the RR of P3AT is sufficiently high [29]. This effect is attributed to better optical absorption and charge transport resulting from the more ordered chain packing of P3HT [30]. However, a high RR is not the only way to achieve high photovoltaic performance—MW [31,32,33], polydispersity (PDI) [34], and ending groups [35] also significantly affect device performance. Notably, the alkyl side-chain plays a principal role in determining the thermal properties and surface tension of the polymers. The side-chain length can directly affect the solubility of P3ATs in solution, thus causing the formation of interchain orders and stacking in the deposited films. Some recent reports have shown that the physical properties and device performance depend on the alkyl substituents [36,37]. However, this evidence is not conclusive because P3ATs with diverse MWs and PDIs were used often under various conditions, which can influence the physical properties of the devices and make their comparisons difficult.

The MW of P3ATs may affect their optical properties [38], solid-state morphologies [31,39], crystallinity [40] and hole mobility [41], and therefore, the physical and photoelectric properties of the P3AT-based devices. However, various trends of MW dependence have been observed, probably because of various experimental conditions and the complexity of the studied object; therefore, conflicting MW effects in conjunction with the side-chain effects on device performance have been reported. Liu et al. [42] reported device efficiency as a function of P3HT MW in the range of 5–48 kDa and found the highest power conversion efficiency (PCE) to be 24 kDa. Spoltore et al. [31] showed a decrease in PCE when the MW increased from 45 to 81 kDa. In contrast, Holmes et al. [43] reported six P3HTs with different MWs (5 to 72 kDa), in which an increase in performance was observed with an increase in MWs for the annealed devices. The conflicting MW criteria for optimal OPV performance indicate that the effect of the MW is still neither accurately estimated nor understood. In addition, the effect of MW on device performance has rarely been reported for P3ATs other than P3HTs.

A systematic understanding of the effects of both alkyl chain lengths and MW is particularly important for improving the efficiency of OPV devices and understanding the complex photoelectric mechanism in these solid systems. Because studies that cover the above-mentioned issues, from synthesis to device efficiency, over various background characterizations with a high degree of completeness are still difficult to find in the literature, we decided to perform a controlled synthesis of P3HT and P3OT with various MWs by adjusting the amount of the Ni(dppp)Cl_2_ catalyst. Additionally, fractions of P3BT with different MWs were obtained via solvent extraction in this study. The effect of MW was accurately investigated by performing a systematic comparison of a wide MW range between the gel permeation chromatography (GPC) MW and the predicted MW. The effect of MW on the thermal properties, particularly on the crystallinity of the various P3AT solids, was investigated by differential scanning calorimetry (DSC). UV–Vis spectroscopy of the P3AT solids was performed using dilute solutions, pristine films, and acceptor blend films of well-defined MW. These studies were carried out in conjunction with photoluminescence (PL) studies and PL quantum yield (PLQY) experiments. Furthermore, the thin films were annealed to investigate their phase behaviors by changing their MWs and side-chain lengths. Finally, P3ATs with various MWs and side-chain lengths were prepared as bulk heterojunction OPV devices to explore their dependence on the device parameters.

## 2. Materials and Methods

### 2.1. Materials

All manipulations were performed in an argon atmosphere using the Schlenk technique. Tetrahydrofuran (THF) was dried over Na/benzophenone and freshly distilled prior to use. The solvents were purchased from Sigma-Aldrich Co. (Saint Louis, MO, USA) and Samchun Pure Chemical Co. (Pyeongtaek-si, Korea) and used without further purification. Isopropylmagnesium chloride solution (2.0 M in diethyl ether) was purchased from Sigma-Aldrich Co. and titrated before use. The catalyst [1,3-bis(diphenylphosphino)propane]dichloronickel(II) and other materials were purchased from Sigma-Aldrich Co. and used as received.

### 2.2. Measurements

^1^H-NMR spectra were recorded on a 400 MHz FT-NMR spectrometer (Bruker, Billerca, MA, USA) at room temperature. ^1^H-NMR data are reported in parts per million (ppm) as chemical shift values relative to tetramethylsilane (TMS) as the internal standard. All NMR spectra were recorded in CDCl_3_. GPC data were recorded using an HP-1047A system (Hewlett Packard, Palo Alto, CA, USA). High performance liquid chromatography (HPLC)-grade THF was used as the eluent, which was maintained at 40 °C. The flow rate was 2.0 mL/min, and the injection volume was 30 μL. The calibration was based on polystyrene standards obtained from American Polymer Standards Co. All polymer samples were dissolved in HPLC-grade THF. DSC curves were obtained using a DSC N-650 instrument (Scinco, Seoul, Korea). All the samples were first heated to 300 °C for 10 min to eliminate their thermal history and then cooled to room temperature. The heating and cooling scans were performed at a rate of 10 °C/min.

### 2.3. General Method for the Synthesis of P3AT

A 500 mL round-bottomed flask equipped with a three-way stopcock was heated under reduced pressure and then cooled to room temperature under an argon atmosphere. 2-Bromo-3-alkyl-5-iodothiophene (26.8 mmol) in dry THF (100 mL) was transferred to the flask using a syringe. Isopropylmagnesium chloride (13.4 mL, 2 M) was added to this mixture using a syringe at 0 °C. A suspension of Ni(dppp)Cl_2_ in dry THF (60 mL) was added to the mixture using a syringe at 0 °C, and the mixture was stirred at room temperature for 48 h. After polymerization was completed, water (200 mL) was added, and the mixture was separated via extraction with CHCl_3_. The organic layer was washed with water and concentrated under reduced pressure. The insoluble material was Soxhlet-washed with MeOH (6 h), acetone (6 h), and hexane (12 h). The P3ATs were collected from the CHCl_3_ fraction by suction filtration to obtain rr-P3AT. The polymers were dried overnight at room temperature under a vacuum. To determine the Grignard exchange reaction time, 5 mL of each solution was taken using a syringe after 10, 20, 40, and 60 min from the addition of isopropylmagnesium chloride and subsequently quenched with water and extracted with ether. The solvent was evaporated, and the residues were investigated using ^1^H-NMR spectroscopy.

#### 2.3.1. Controlled MW P3BT Polymer

After washing with hexane, three different MW rr-P3BT fractions were Soxhlet extracted with CHCl_3_ every 6 h. ^1^H-NMR: δ 6.98 (s, 1 H), 2.83 (t, 2 H), 1.74 (quint, 2 H), 1.45 (m, 2 H), 1.35 (m, 4 H), 1.00 (t, 3 H).

#### 2.3.2. Controlled MW P3HT Polymer

The MW of rr-P3HT was controlled by the amount of Ni(dppp)Cl_2_ catalyst (ratios (mol/mol) of [**1b**]_0_/[Ni]_0_ from 71 to 1000). ^1^H NMR (400 MHz, CDCl_3_): δ 6.98 (s, 1 H), 2.80 (t, 2 H), 1.71 (quint, 2 H), 1.44 (m, 2 H), 1.35 (m, 4 H), 0.93 (t, 3 H).

#### 2.3.3. Controlled MW P3OT Polymer

The MW of rr-P3OT was controlled by varying the amount of Ni(dppp)Cl_2_ catalyst (ratios (mol/mol) of [**1c**]_0_/[Ni]_0_ from 68 to 340). ^1^H NMR (400 MHz, CDCl_3_): δ 6.98 (s, 1 H), 2.82 (t, 2 H), 1.72 (quint, 2 H), 1.43 (m, 2 H), 1.31 (m, 4 H), 0.90 (t, 3 H).

### 2.4. UV-Visible Absorption and Photoluminescence Studies

A two-beam UV–Vis spectrometer (Neosys-2000, Scinco) was used to obtain the UV–Vis absorption spectra of the solution and thin film samples. Steady-state PL spectra for both the solution and film samples were measured using a spectrophotometer (FS-2, Scinco), and absolute PLQYs were obtained using a commercial system (Quantaurus-QY, C11347, Hamamatsu Photonics K.K., Hamamatsu, Japan) equipped with an integrating sphere. The concentration of the solution samples used for recording the UV–Vis spectra was approximately 1 × 10^−4^ weight% (wt%) and 1 × 10^−5^ wt% in chlorobenzene. Notably, the solution samples were prepared using wt% to maintain a consistent quantity of polymers in each solution rather than using molar concentration due to the MW distribution of the polymers. A solution with a relatively low concentration was used to record the PL spectra to avoid any severe concentration effects and to depress any significant reabsorption features. To maintain consistency, the films were prepared using conditions similar to those used in the preparation of the active layers within the solar cell devices, which are discussed in the next sub-section.

### 2.5. Fabrication and Characterization of Polymer Solar Cells

The polymer-based bulk heterojunction OPV devices were fabricated as follows: pre-patterned ITO-covered glass substrates (Samsung Corning, Asan-si, Korea) were ultrasonically cleaned for approximately 15 min in deionized water with <2% detergent (Mucasol, Sigma-Aldrich Co.), deionized water, acetone, and isopropanol. The substrate was then treated with oxygen plasma for 10 min. A poly(3,4-ethylenedioxythiophene) polystyrene sulfonate (PEDOT:PSS, Clevios P VP AI 4083, Heraeus Clevios, Leverkusen, Germany) layer with a thickness of 40 nm was spin-coated at 3200 rpm onto the pre-treated ITO-covered glass substrates and then baked at 150 °C for 15 min under ambient conditions. A 2 wt% chlorobenzene P3AT: phenyl-C_61_-butyric acid methyl ester (PCBM, Nano-C) solution with a 1: 0.7 weight blending ratio was stirred for 1 h at 60 °C and then at room temperature overnight. P3HTs with various MWs (15, 21, 39, 52, and 72 kDa) and all synthesized P3OTs (12, 13, 18, 38, 72 kDa) were used for device fabrication. The prepared blend solutions were filtered through a 0.45 μm syringe filter and then spin-cast onto the PEDOT: PSS-covered ITO substrate. Subsequently, a film thicknesses of approximately 90 nm at a spin-coating speed of 1300 rpm was obtained. To obtain the top electrode, 0.3 nm lithium fluoride and 120 nm aluminum were thermally evaporated under a 10^−6^ mbar vacuum. To study the effect of annealing on the device performance, each device was encapsulated by an engraved cover glass in addition to a commercial getter under N_2_ atmosphere, followed by post-annealing at 150 °C for 15 min. In general, the prepared device structure was ITO/PEDOT:PSS (40 nm)/P3AT:PCBM (90 nm)/LiF (0.3 nm)/Al (120 nm). The current density-voltage (J-V) characteristics of the P3AT-based OPV devices were measured using a 2410 source-measuring unit (Keithley, Solon, OH, USA) under the illumination of a 94062A solar simulator (Newport, Irvine, CA, USA) in a clean room kept slightly below 25 °C. To maintain the conventional standard test conditions, i.e., air mass of 1.5 as spectral irradiance distribution and 1000 W/m^2^ as radiant intensity, the solar simulator used was calibrated using reference cells certified by the National Renewable Energy Laboratory (NREL, Golden, CO, USA). The external quantum efficiency (EQE) spectra were obtained using commercial equipment (QEX7, PV Measurement Inc., Boulder, CO, USA). Notably, a blending ratio of 1:0.7 was chosen for comparison with the optimized device parameters obtained using commercial P3HT. Our previous study focused on the blending ratio-dependent device performance and showed that the optimum blending ratio to obtain a statistically reliable and relatively high PCE of >4% was approximately 1:0.7 [44].

## 3. Results and Discussion

### 3.1. Synthesis and MW Distributions of P3AT

**Scheme 1:**
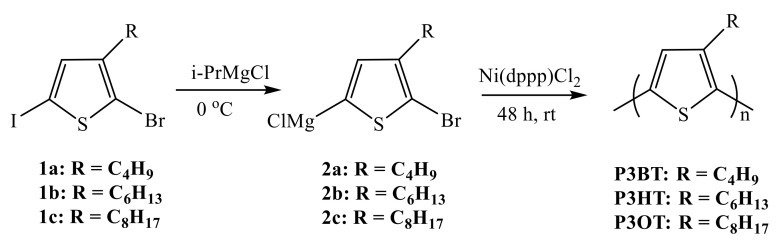
Synthesis route of rr P3AT.

GRIM polymerization, a popular method that enables control of the MWs, PDIs, and RR of P3AT under relatively mild conditions, is a living chain growth process rather than a step growth process [45,46]. In this method, the Grignard reagent exchange reaction was first carried out between 2-bromo-3-alkyl-5-iodothiophene and one equivalent of isopropyl magnesium chloride; for example, the synthesis of P3HT with a reaction time of 20 min was performed at a small scale (<1.5 mmol of monomer) [47]. However, in the present work, a considerably greater amount of monomer was used to fabricate the polymer, which can lead to a longer reaction time. To obtain the optimal conditions, the conversion of **1b** at different periods was conducted using the ratios of 2-bromo-3-hexylthiophene generated in subsequent quenching with water to monomer **1b** by ^1^H-NMR spectroscopy (Figures S1 and S2). Compared to the small-scale synthesis of P3HT, the Grignard exchange reaction requires a longer reaction time. As shown in Figure S1, the conversion of **1b** was only 82% within the first 20 min, and the reaction was completed after 1 h. To ensure standard conditions for controlling the MW polymerization of P3AT, Ni(dppp)Cl_2_ was added to the reaction after 2 h. Subsequent polymerization was carried out within 48 h at room temperature (Scheme 1). The resulting P3HTs were purified by Soxhlet extraction with methanol, acetone, and hexane in succession to completely remove the residual monomer and oligomer.

To determine the MW of P3ATs, conventional methods, such as matrix-assisted laser desorption ionization-time-of-flight (MALDI-TOF), ^1^H-NMR, and GPC, can be efficiently employed. Matrix-assisted laser desorption-ionization (MALDI) and NMR MW have been documented more closely to the real mass of polythiophenes in the low-MW region. However, both MWs are limited in the high-MW region owing to the low resolution of the signal peaks. Thus, GPC measurements are important for determining the high MWs of polythiophenes. However, systematic errors in the GPC measurements using polystyrene standards have been observed for two reasons: (1) structural differences between P3HT (rod-like) and polystyrene standards (coil-like) [48] and (2) different desorption efficiencies of the polymers in the GPC column, leading to different retention times of the polymers [49]. The systematic errors in GPC measurements of the MWs of polythiophenes are generally hypothesized to be MW overestimations [49]. However, it should be noted that the aforementioned factors are provided for low-MW regimes (<30 kDa). Therefore, a standard value in a high MW range is necessary to determine the diverse MWs of polythiophenes by GPC measurements.

**Figure 1:**
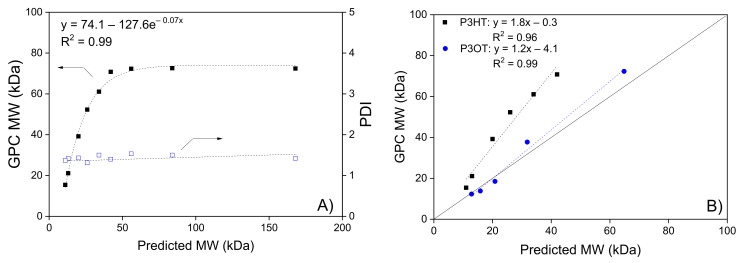
GPC MW versus predicted MW calculated using the [monomer]_0_/[Ni]_0_ ratio: GPC MWs and PDIs of (**A**) P3HT and (**B**) P3HT (15–70 kDa) and P3OT (12–72 kDa).

Two series of polymers, P3HT and P3OT with various M_n_, were synthesized by adjusting the feed ratios of monomer **1** to the Ni catalyst ([monomer]_0_/[Ni]_0_), while MW fractions of P3BT were obtained using the solvent extraction method. Related data are presented in Table 1. The M_n_ values of P3HT and P3OT were determined using two conventional methods: GPC and ^1^H-NMR. The ^1^H-NMR analysis based on the integrals of the main-chain and chain-end protons, which appear at signals of 2.85 and 2.65 ppm, respectively, can provide a general estimate of the P3AT MW [50]. Although the NMR M_n_ of P3AT could not be determined accurately at high ratios of [monomer]_0_/[Ni]_0_ (more than 330), the relationship between the MWs of P3AT and the ratios is linear, as shown in Table 1 and Figure S3. Seferos et al. [49] demonstrated that all NMR M_n_ of P3HTs agreed well with the predicted M_n_ calculated with the corresponding ratios of [monomer]_0_/[Ni]_0_. The predicted M_n_ and GPC M_n_ values were compared by plotting the actual GPC values as functions of the predicted values, as shown in Figure 1.

Previous reports have shown that GPC measurements mostly yield P3HT MWs higher than the real values. Thus, M_n_ calculated by GPC is 1.2–2.3 times larger than that calculated from the MALDI-TOF results depending on the different MW fractions [27]. A similar observation was reported by two research groups that provided coefficients of 1.3 [49] or 1.6 [50] for the deviation between the GPC MW and real mass for the low-M_n_ regime (less than 23 kDa). The results are shown in Figure 1. It is clear that the GPC MW of the resulting P3HTs is an exponential function of the M_n_ calculated by catalytic ratios with an asymptote value of 74.1 ± 1, as shown in Figure 1A. This could be explained by the poor solubility of the high-MW regime in the reaction solvent THF, which causes aggregation of the polymer during polymerization (Table S1). A similar phenomenon was observed in the GPC measurements at 140 °C. However, the GPC M_n_ obtained in the present study increased faster than that in a previous report [50]. In this study, with GPC operated at 40 °C, a linear relationship between the predicted M_n_ and GPC M_n_ is observed in a wide range of MWs between 15 and 70 kDa, where an overestimation occurs by a factor as high as 1.8 ± 0.1 (standard error), as shown in Figure 1B. This may be because high-MW regimes have poor solubility in THF at low temperatures, leading to a reduction in the absorption efficiency of the polymer in the column, which leads to faster elution from the column.

Similarly, a series of P3OTs with various MWs, which possess higher solubility than P3HT in THF at a comparable MW, was controlled by adjusting the ratio of [monomer]_0_/[Ni]_0_. In addition, the comparison of the two MW values calculated by catalytic rates and GPC shows that the MW value from the GPC data systematically overestimated the predicted MW. Importantly, the MW of P3OT calculated by GPC was only 1.2 ± 0.1 (standard error) times greater than the predicted MW, although the MW range was determined to be similar to that of P3HTs (12–72 kDa). This indicates that the GPC MW measurement for P3OT yield values were closer to the predicted MW for P3HT. In other words, this result indicates that P3OT was absorbed better in the GPC column than P3HT under the same conditions. We conclude that the solubility of polythiophenes in GPC is an important factor that can be used to control the standard error parameters in GPC measurements to determine the true MW of polythiophenes.

Additionally, GPC analysis revealed that most polymers possessed a narrow polydispersity (1.3–1.5 PDI) even for high-MW polymers. This confirms that the living chain polymerization mechanism can be performed at a very low termination reaction rate. The RRs of all P3ATs estimated from ^1^H NMR data (Figure S4) are approximately 98–99%, as determined by comparing the protons at the 2-position of the thiophene ring, which appear in the range of 6.98–7.00 ppm [51]. Notably, an improved performance of the OPV devices can be expected when P3ATs with such high RRs are used. To evaluate the effect of MW on the physical and photoelectrical properties of polymers, P3HTs, P3BTs, and P3OTs covering a wide range of MW values were used for further studies, which has been described in the following sections.

### 3.2. Physical Properties

#### 3.2.1. Thermal Properties

**Figure 2:**
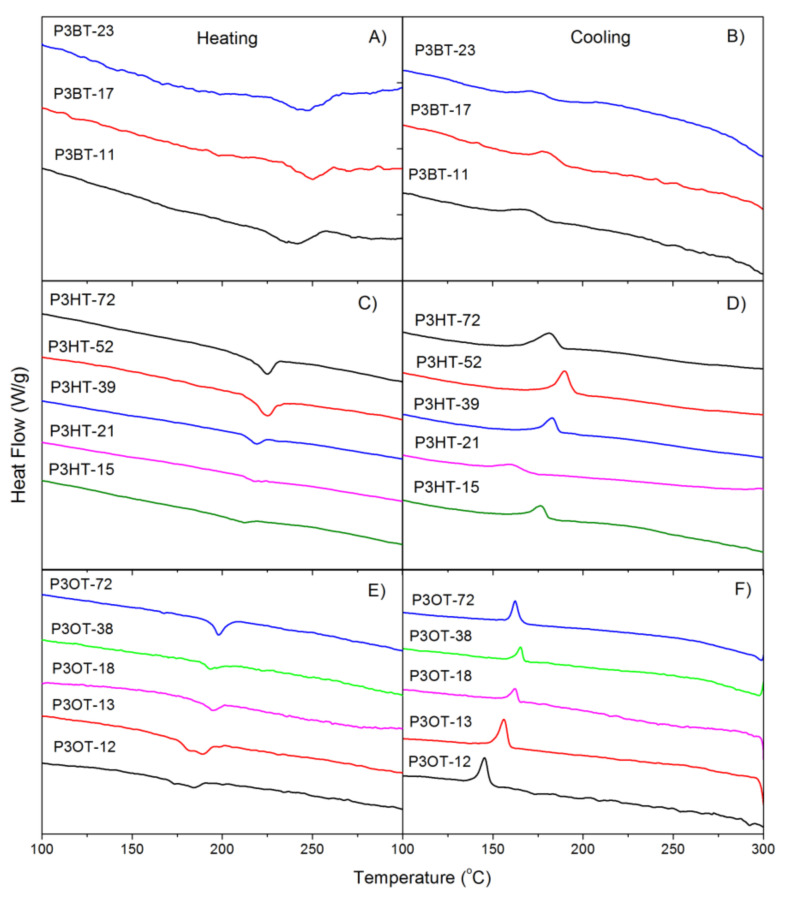
DSC of heating (left) and cooling (right) curves of the samples with different MWs: P3BTs (**A**,**B**), P3HTs (**C**,**D**), and P3OTs (**E**,**F**). Heating rate 10 °C/min. N_2_ atmosphere.

DSC was used to evaluate the thermal properties and crystallinity of the P3ATs. The second heating and cooling traces for the various MWs of the P3AT samples are shown in Figure 2. The broadest endothermic peaks were observed for P3BTs, explaining the highest corresponding values of enthalpy of fusion (ΔH_f_) (Table S2). For the synthesized P3HTs, the heating traces of the low-MW samples, P3HT-15 and P3HT-21, feature small endothermic peaks. Interestingly, the heating traces of the low-MW P3OTs show two distinct endothermic peaks, while those of the high-MW samples, P3OT-38 and P3OT-72, each display only a single endothermic peak at 194 and 198 °C, respectively.

**Figure 3:**
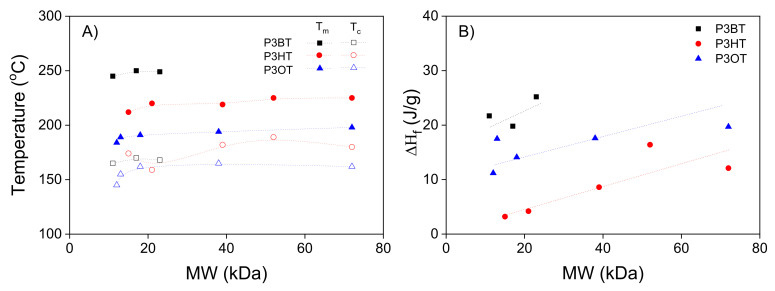
Plots of (**A**) crystalline melting temperature (T_m_), crystalline temperature (T_c_), and (**B**) enthalpy of fusion (ΔH_f_) versus MWs of P3AT.

**Figure 4:**
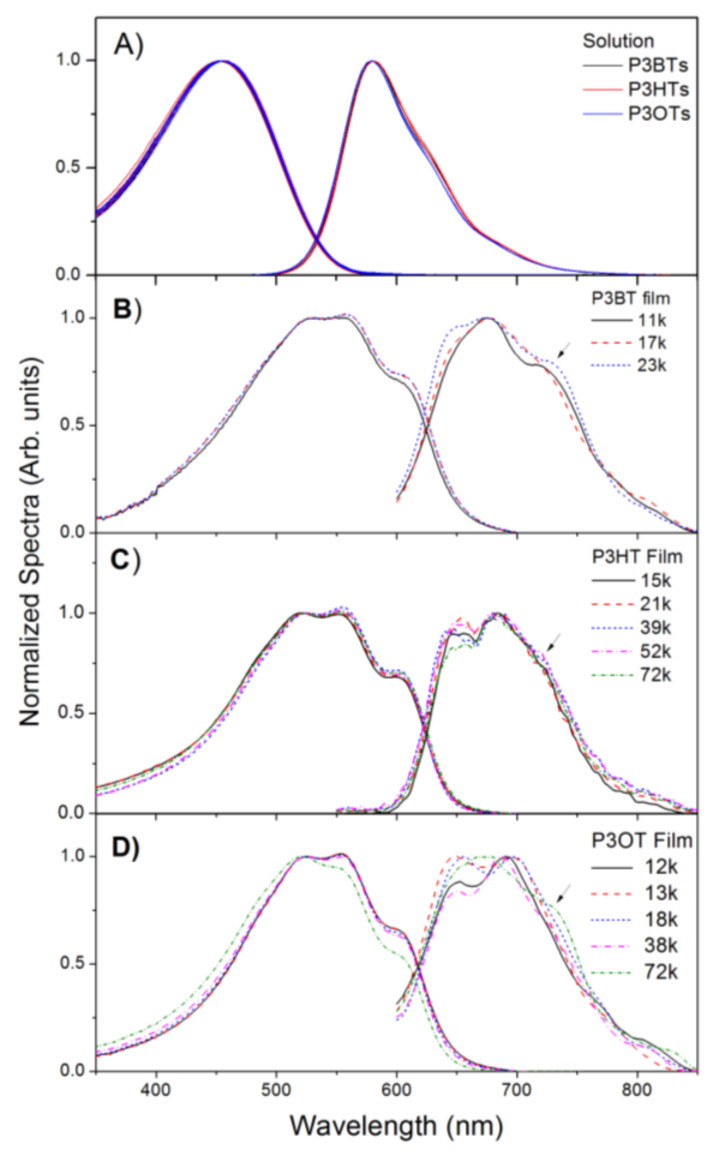
UV–Vis & PL spectra: (**A**) diluted solutions of all P3ATs with various MWs and non-annealed pristine films of (**B**) P3BTs, (**C**) P3HTs, and (**D**) P3OTs with various MWs.

The crystalline melting temperature (T_m_), crystalline temperature (T_c_), and enthalpy of fusion (ΔH_f_) of the P3ATs with various MWs are presented in Figure 3. A slow increase in T_m_ values with increasing MW for all P3ATs is observed in Figure S5. T_m_ is affected by both chain packing and crystal size, as reported in the literature [42]. Therefore, the slow increase in T_m_ with MW reveals that the effect of MW on chain packing and crystal size is not very high; otherwise, these properties are similar for polymers of the same type. The T_m_ values of P3AT are in the order of P3BT > P3HT > P3OT. This leads to a significant difference in chain packing and crystal size between the different P3ATs. This observation is in agreement with an earlier report [52]. In addition, Figure 4A shows T_c_ as a function of P3AT MW and the highest values achieved at medium MWs (P3BT-17, P3HT-52, and P3OT-38).

The crystallinities of P3ATs can be calculated using their melting enthalpies with the equation X = ΔH_f_/ΔH_f_^o^, where X, ΔH_f_, and ΔH_f_^o^ are the crystallinity, enthalpy of fusion, and enthalpy of fusion of ideal crystalline P3AT, respectively [53]. Enthalpy of fusion of P3ATs was calculated from the area under the endotherm in Figure 2A,C,E, and the data are shown in Table S2 and Figure 3B, where the highest values are observed for P3BT-23, P3HT-39, and P3OT-72, 25.2, 16.4, and 19.7 (J/g), respectively. The calculated enthalpies of fusion for 100% crystalline P3HT and P3OT were 99 and 77 J/g, respectively; [54] therefore, the degree of crystallinity of P3HTs ranged from 3.2 to 16.6%, while that of P3OTs ranged from 14.5 to 25.6%.

Although the enthalpy of fusion of ideal crystalline P3BT (ΔH_f_^o^), which is a constant for a polymer type, has not been reported in the literature, the enthalpy of fusion or crystallinity of all P3ATs generally increases with the increase in MW, as shown in Figure 3B. This is in agreement with a previous report [55] where attributed crystallites were formed from a small number of chains with sufficient molecular length by the stacking of the extended chains.

#### 3.2.2. Optical Properties

The UV–Vis and PL spectra of the diluted solutions and pristine films of all P3ATs with various MWs are shown in Figure 4. The maxima of the UV–Vis spectra originate from the π-π* transitions, which are typical for the π-conjugated P3ATs, as discussed in previous studies [37,41,56]. The first common tendency is that both the UV–Vis and PL spectra of the films are significantly red-shifted to those of the solutions after solidification. The maxima of the solution UV–Vis spectra centered at ~454 nm are red-shifted to ~523 nm, as ∆E~0.36 eV, while the maxima of solution PL centered at ~580 nm are red-shifted to ~652 nm, as ∆E~0.24 eV. Interestingly, the degrees of the red shifts were virtually unaffected by the side-chain lengths of all the pristine films. In addition, well-resolved fine peaks were found in the UV–Vis spectra of all pristine films, which is known to be a sign of the pronounced interchain interaction-induced aggregation ability of the rr-polythiophenes, which has been reported in detail elsewhere [44,56]. If one takes the energetically lowest shoulder peak centered at ~602 nm, the red-shift is up to ~0.67 eV. The amplitudes of the most red-shifted bands at 602 nm can be compared with those of the less interchain interaction-induced, therefore, only weakly red-shifted bands to avoid severe overlapping.

All films exhibited pronounced fine structures in the PL spectra, which can be assigned to the C=C double bond stretching mode of molecular vibration [56]. More specifically, the PL bands centered at ~650 nm, ~680 nm, and ~730 nm were assigned as 0-0, 0-1, and 0-2 vibronic transitions, respectively [36,37]. It is noteworthy that the PL spectra of P3ATs showed a clear side-chain dependence on 0-2 vibronic transition at peak ~730 nm. Indeed, P3BTs exhibited the pronounced shoulder while a weak peak at this position appeared in the PL spectra of P3HTs however, no signal in the PL spectra of P3OTs was observed, except for P3OT-72. It seems that the PL intensity of the vibronic peak, especially centered at ~730 nm, increased with a decrease in the alkyl side-chain length. Similar side-chain length-dependent PL behaviours were observed by Thankaraj Salammal et al. [36] when the films were casted at room temperature. However, they have found that the side-chain length dependency was diminished when the films were casted at −30 °C. They attributed to the side-chain length dependency as the degree of the crystallinity of the films because the crystallinity increased upon lowering the cast temperature. In addition, it might be suggested that the poor interchain stacking of the P3AT backbone with a longer side-chain length caused by the flexibility of the alkyl side-chain might be responsible for the PL dependency. Previous reports [57,58] have shown that the torsion of the thiophene backbone is caused by torsion of the alkyl side-chains. This can lead to an increase in the plasticity of the backbone, which subsequently loses its coplanarity with the adjacent thiophenes. Therefore, a shorter side-chain length can reduce the disorder in the film, leading to an increase in the luminescence intensity at the shoulder peak centered at ~730 nm.

**Figure 5:**
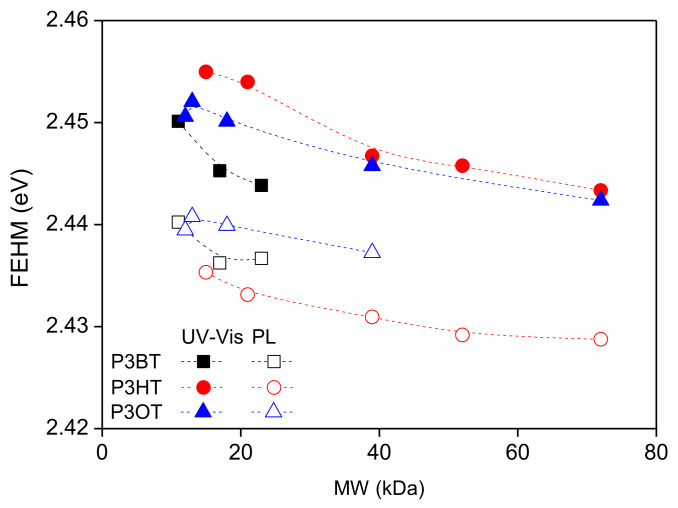
Plots of the FEHM of UV–Vis and PL spectra measured with the diluted solutions of P3ATs against MW. Dotted lines are shown only for visual guidance.

The estimated front edge values at half maxima (FEHM) of the UV–Vis and PL spectra of the diluted solutions as functions of MW are shown in Figure 5. The positions extracted from the FEHM values are marked in the spectra shown in Figure S6. Interestingly, the red-shift trend that appeared in the PL spectra was comparable to that in the UV–Vis spectra of the diluted solutions. All three side-chains, butyl, hexyl, and octyl, showed a clear increasing red-shift effect with increasing MW. It seems that the pristine films also exhibit similar MW dependencies. However, this feature was not clearly verified because of the presence of pronounced interchain interactions in the films. This red-shift in the UV–Vis spectrum of the dilute solution might be regarded as a measure of the effective conjugation length (ECL) of the conjugated macromolecules to explain the limit of the red shift of long polymer chains despite the marginal degrees of the red shifts [59,60]. The ECL can be used to discuss the number of monomer units that behave as a joint sub-unit in an optical transition process. To estimate the ECL, most previous studies have used model compounds with relatively short chains and well-defined structures, such as oligomers ranging from trimers to dodecamers [54]. This result is crucial because polymers with chains significantly longer than the previously reported 10–15 monomer units still exhibit red shifts. However, the practical value of ECL must be shorter than the entire length of the P3ATs because the probability of attaining coplanarity for smooth conjugation along the long polymer backbones must decrease with increasing MW. The marginal differences in red shifts reflect the fact that the red shift must be proportional to the reciprocal values of the ECL. These values are often expressed by the number of closely bound monomers on a polymer chain, which can be significantly greater than those of the oligomeric model compounds. The virtually side-chain length-independent UV–Vis spectra of dilute solution samples reveal that the main electronic transition must be related to the π-π* transition. These transitions could be caused by the thiophene units on the conjugated polymer backbones rather than the side-chains, particularly in such a less interchain interaction-pronounced situation.

**Figure 6:**
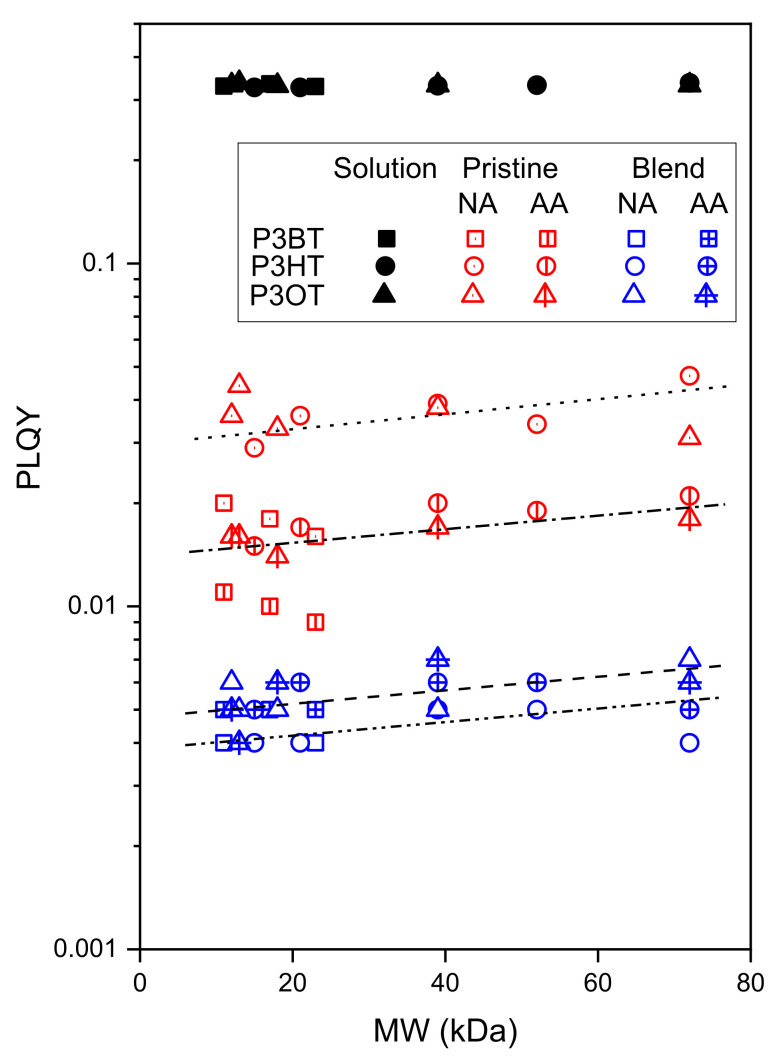
PLQYs of diluted solutions and pristine and blended films. All the lines in the plot are for visual guidance: the dotted line represents the non-annealed pristine films, the dash-dotted line represents the annealed pristine films, the dashed line represents the annealed blend films, and dashed-dotted line represents the non-annealed films. NA and AA stand for non-annealed and after-annealed conditions, respectively.

All PLQYs estimated in this study are presented on a logarithmic scale (Figure 6). The estimated PLQYs of most solutions were 0.33. This is understandable because the main radiative channel might be the π*→π transition; therefore, the alkyl side-chains cannot considerably affect fluorescence during the depressed interchain interactions. However, the PLQYs of the films are dramatically affected by the blending and preparation history of their solid states. The average PLQY of the non-annealed pristine films was approximately 0.035, which is approximately an order of magnitude lower than that of the solutions. After annealing, the PLQY of the pristine films was reduced to 0.017, which is approximately half that of the non-annealed pristine films. An exception is observed in the case of P3BTs because their average PLQYs were 0.018 and 0.010 for the non-annealed and annealed samples, respectively. This means that P3ATs with short side-chains should have higher exciton diffusivity toward quenching sites or a larger population of physical defects, leading to effective PL quenching [55].

Interestingly, the average PLQYs of the blended samples were 0.0057 and 0.0046 for the annealed and non-annealed samples, respectively. The difference of the blended samples was not significant, while the pristine films showed a significant reduction in PLQY upon annealing. In addition, the P3BTs are not an exception to this trend, as shown by the values of the pristine samples. Hence, the PLQYs of the blended films have the lowest values, which are approximately two orders of magnitude lower than those of the solution samples. It is noteworthy that the values for most P3ATs increase slightly with an increase in MW. For P3BTs, the MW dependency could not be confirmed because the data on the P3BT samples with sufficient chain lengths were not available to verify this effect.

**Figure 7:**
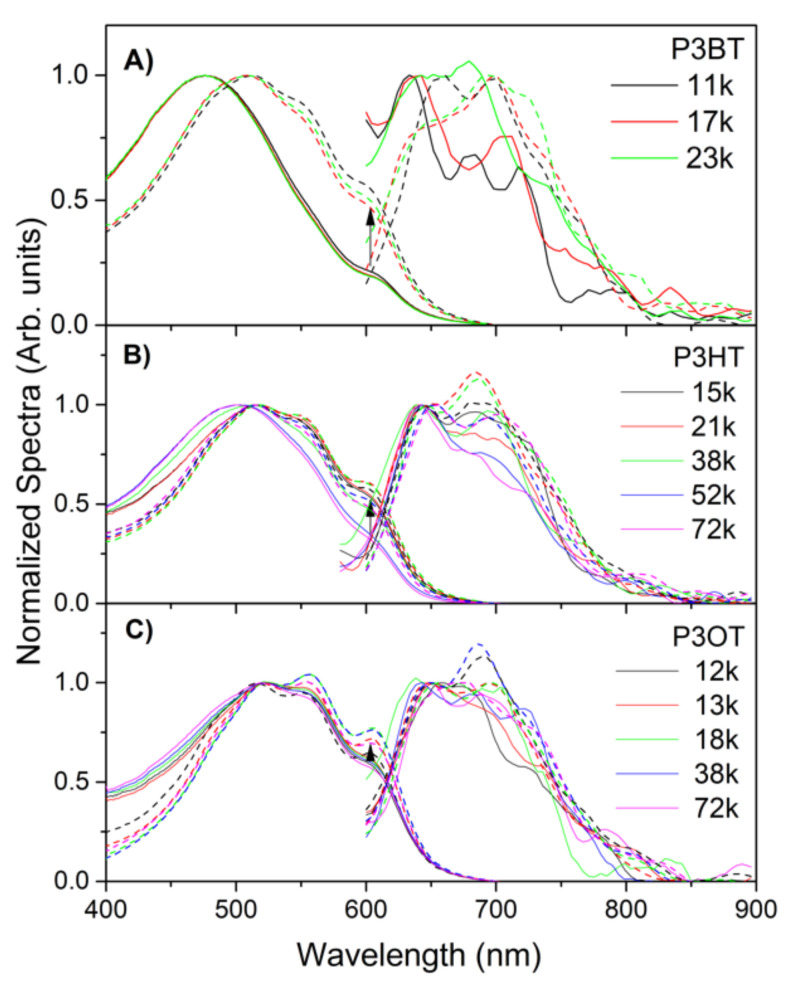
UV–Vis and PL spectra of the blended films of (**A**) P3BTs, (**B**) P3HTs and (**C**) P3OTs. Solid plots represent the non-annealed and dashed plots, the annealed samples. Arrows are used to show the amplitude changes in the bands centered at 602 nm upon annealing.

The UV–Vis and PL spectra of the blended P3AT films are shown in Figure 7. The P3BT-blended films exhibited a virtually structureless UV–Vis spectra, except for the small shoulder peaks centered at ~602 nm. P3HT blended films with longer MWs (52 kDa and 72 kDa) also showed relatively weak peaks centered at ~602 nm, while those with shorter MWs (15, 21, and 38 kDa) show pronounced peaks at ~602 nm. However, the spectra of all P3OTs showed pronounced shoulder peaks at ~602 nm, even before annealing. The heights of all weak peaks at ~602 nm increased after annealing and became comparable to those of the pronounced peaks before annealing.

Interestingly, the maxima of the UV–Vis spectra before annealing exhibited a similar red-shifted trend, analogous to that of the peak heights at ~602 nm. Moreover, the maxima of the P3BTs exhibited a weak red-shift compared to the mutual peak positions of solutions ranging from 454 nm to 500 nm. The heights of the weak peaks at ~602 nm and the corresponding red-shifts of the maxima significantly depended on the side-chain lengths and were in the order of P3BTs < P3HTs < P3OTs; however, these dependencies diminished after annealing. Interestingly, the spectral positions of the three peaks at ~602 nm, ~555 nm, and ~522 nm caused by film formation were virtually unaffected by the MWs, side-chain lengths, annealing, and blending. Notably, the features that the spectral positions of the bands at 602 nm with side-chain length-dependent amplitudes were unchanged upon annealing, although their peak amplitudes converged towards to a similar value upon annealing, and the side-chain length-dependent red-shifts of the maxima were further red-shifted but also converged towards a similar value of ~520 nm, have exactly the same tendency upon annealing. These reveal that the UV-Vis bands at 602 nm is the newly formed interchain interaction-induced bands while the less red-shifted maxima must belong to the bands of the chains which cannot properly interact before annealing because they were further red-shifted, or reduced, as the peak amplitudes of the bands 602 nm increased upon annealing.

**Figure 8:**
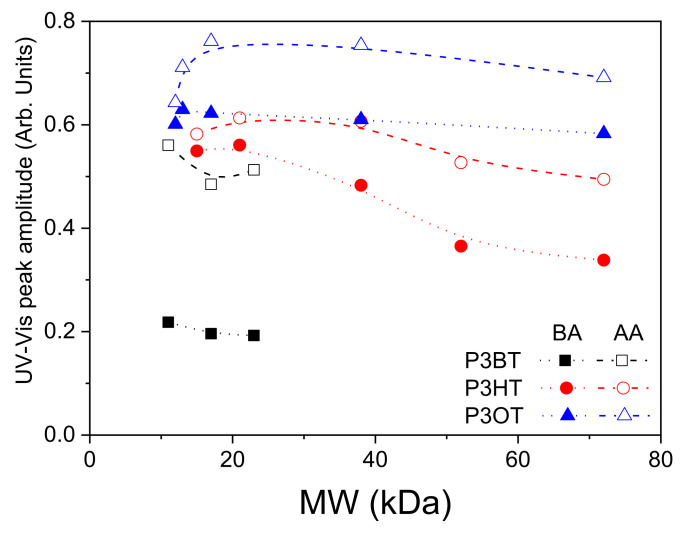
Plots of the peaks of the blended films centered at 602 nm as functions of MW. Values were extracted from the UV–Vis spectra shown in Figure 7. (BA = before annealing, AA = after annealing).

The relative peak heights of the bands at ~602 nm seem to be MW-dependent, but not in a simple linear manner. To analyze this phenomenon, the relative peak values were plotted as a function of MW in Figure 8. Interestingly, P3ATs with longer side-chains seem to exhibit effective interchain interaction abilities in the blended systems before annealing. This can be attributed to the higher solubility of P3ATs with shorter chains. Therefore, P3ATs with shorter chains may exhibit higher miscibility with the acceptors before annealing. However, after annealing, most of the UV–Vis spectra featured comparable interchain interactions and peak positions, as reported previously [61]. The PL spectra exhibited red-shift features, which were similar to those in the UV–Vis spectra. However, because of the very strong acceptor blending-induced quenching and consequently low PLQYs, the quality of the steady-state PL spectra is insufficient and cannot be discussed in more detail. In addition, more factors for the change in PL behavior should be taken into consideration to better understand such spectral relaxation phenomena.

The relative peak heights of the UV–Vis bands of the blended films at 602 nm were plotted as functions of MW, as shown in Figure 8. Before annealing, the side-chain-dependent aggregation abilities of the films can be clearly verified in the following order: P3BTs < P3HTs < P3OTs. For the P3HTs and P3OTs, the relative heights of the bands increased rapidly with increasing MW, while those of the P3BT bands were not significantly MW-dependent. The plots of height versus MW coincide with the maxima at ~20 kDa for P3OT- and ~30 kDa for P3HT- annealed films. These plot heights were more extended than those of the non-annealed blend cases. Although the MW dependencies of the three P3ATs seem complicated, they became impressively similar and relatively unaffected by the MW after annealing. All P3BTs and P3HTs with longer chains showed less pronounced interchain interaction-induced bands centered at 602 nm before annealing, which indicates that they were homogeneously dispersed in the blend solids. It is crucial that the MW dependencies of the relative peak heights centered at 602 nm before annealing coincide with those of T_m_, as independently obtained by the GPC experiment. However, these differences dramatically decreased after annealing as the relative peak heights became comparable. This observation indicates the possibility of further phase separation, which may result in pronounced interchain interactions. After annealing, the polymer chains became closer to enable stronger interchain interactions, which can be attributed to the PCE improvement.

### 3.3. Electrical Properties

**Figure 9:**
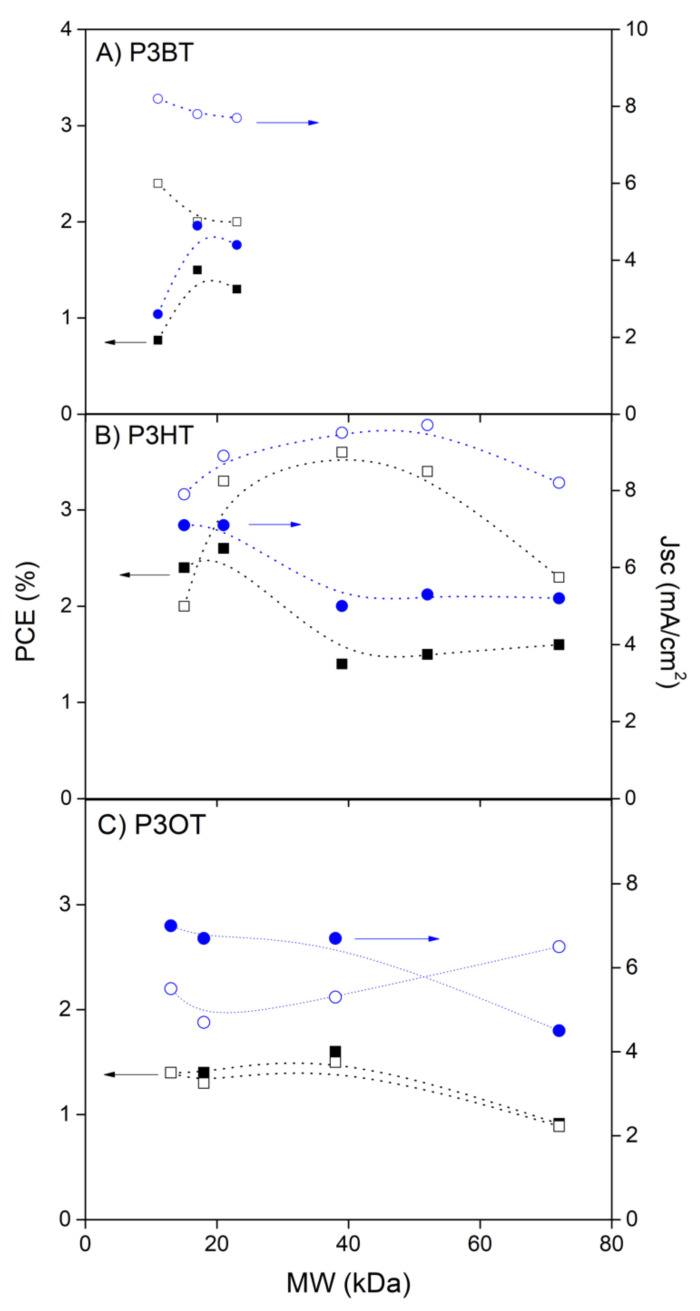
PCE and J_SC_ of all the blended P3ATs before (solid symbols) and after (open symbols) annealing: (**A**) P3BTs, (**B**) P3HTs, and (**C**) P3OTs. Dotted lines are included for guidance.

**Figure 10:**
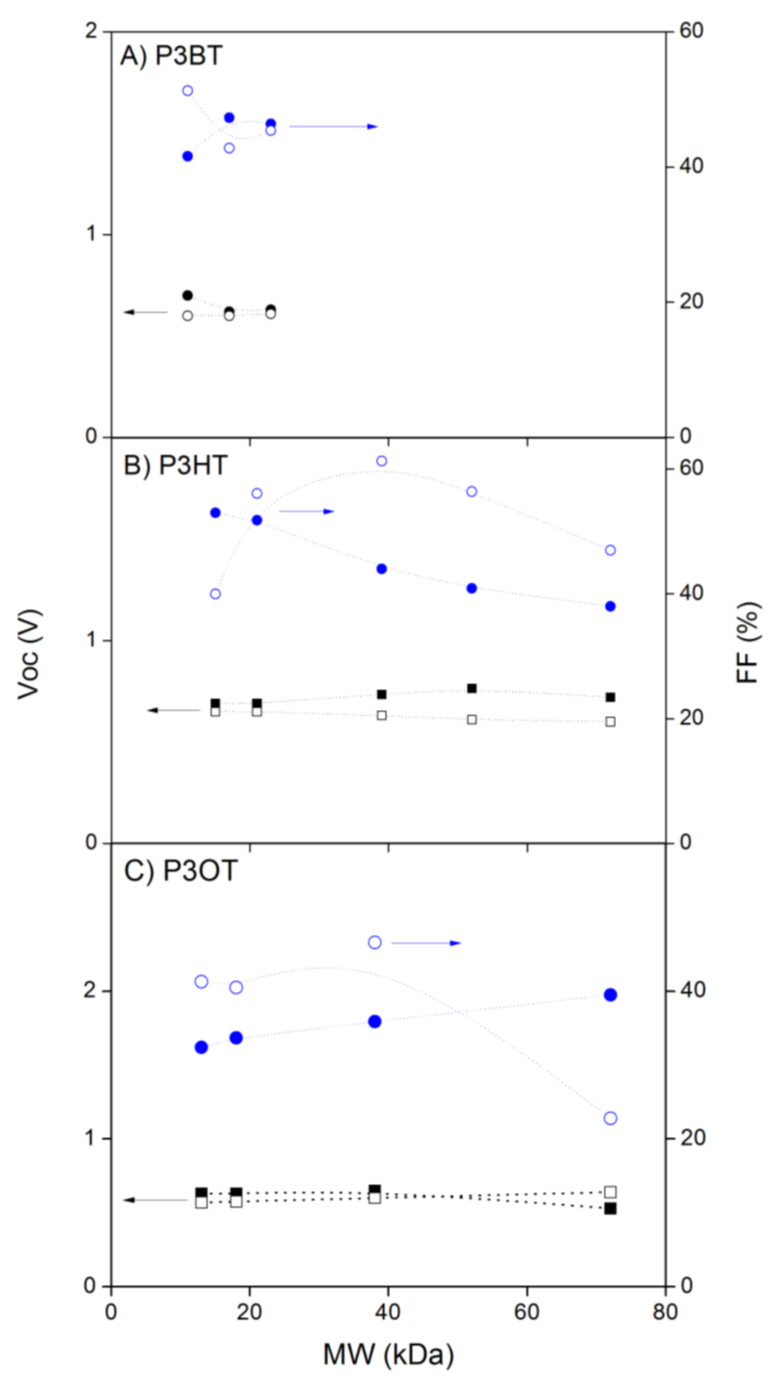
V_oc_ and FFs of all P3ATs before (solid symbol) and after (open symbol) annealing: (**A**) PBTs, (**B**) P3HTs, and (**C**) P3OTs. Dotted lines are for visual guidance.

The J-V characteristics of the devices before and after annealing were measured for all the P3ATs (Figure S7). The most pronounced change upon annealing is the increase in J_SC_ for P3BTs and P3HTs; however, J_SC_ decreases for P3OT after annealing, as shown in Figure 9. Interestingly, this change in J_SC_ is very similar to that of the relative peak heights extracted from the UV–Vis spectra (Figure 8). An increase in fill factors (FF) upon annealing can also be observed, while V_OC_ is not affected by annealing, as shown in Figure 10. The PCE values of the P3OTs before annealing were clearly higher than those of the P3HTs. These results are similar to those obtained from the UV–Vis spectra. Here, the UV–Vis peaks of the P3OTs at 602 nm were more pronounced than those of P3HTs before annealing. This clearly shows that the highly ordered stacking of the P3ATs backbones is a determinant for the PV device performance owing to the enhanced charge carrier mobility caused by the highly ordered stacking rather than charge carrier photo-generation occurring prior to the charge carrier transport to the electrodes.

Before annealing, the highest PCE values were obtained for the following P3AT samples: P3BT-15 (1.5%), P3HT-21 (2.6%), and P3OT-38 (1.6%). Moreover, the highest PCE values of P3BT-11, P3HT-39, and P3OT-38 were 2.4%, 3.6%, and 1.5%, respectively. The electronic efficiency of P3AT-based OPV devices in the present work is comparable to previously reported values [62,63,64]. It might be useful to compare the highest values of the synthesized P3HT devices fabricated in this study with the values obtained with commercial P3HT (Rieke Metal) devices prepared under the same conditions. After annealing, an OPV device with commercial P3HT exhibited a typical PCE of approximately 4.3% with the device parameters of J_SC_ = 10.3 mA/cm^2^, V_OC_ = 0.62 V, and FF = 67.4%, which are approximately 16% higher than those of the synthesized P3HT. This deviation was probably caused by the fact that different synthesis pathways might lead to different MWs, PDIs, and RRs of the commercial P3HT. Moreover, the device preparation conditions were optimized for the commercial P3HTs, but not for the synthesized P3HT, to maintain consistency for better comparison. The related device parameters are listed in Table S3.

The obtained V_OC_ values were approximately ~0.6 V and virtually independent of the side-chain length, MW, and annealing. However, J_SC_ and FF values were found to be dependant on these factors, as shown in Figure 10. The weak dependence of V_OC_ upon the changing MWs and side-chain lengths indicates that the V_OC_ of the OPV device using P3AT mainly depends on the mutual characteristics of the polymer backbone consisting of thiophene moieties. These V_OC_ values are unperturbed by various packing effects, including molecular morphology, crystallinity, solubility, and charge carrier transport ability, unless the blending ratio or active layer thickness of the OPV devices is changed.

P3HTs exhibited impressive PCE improvements upon annealing, especially for MWs of 39 kDa and 52 kDa. However, P3HTs with MWs of 15 kDa, 21 kDa, and 72 kDa did not show such a dramatic increase in PCEs upon annealing. Therefore, the PCE plot as a function of MW shows a maximum of approximately 39 kDa. Although the PCE of P3HT-52 exhibited the highest J_SC_ after annealing, the overall PCE of P3HT-39 was slightly higher than that of P3HT-52 because of its high FF value. In addition, the corresponding V_OC_s do not play a significant role because they are virtually independent of the MW. This situation can be understood with the relation between PCE and the device parameters as described by the following equation: PCE = J_SC_ × V_OC_ × FF. The PCEs of P3BTs with relatively lower MWs also exhibited effective PCE improvements upon annealing, which is attributed to their J_SC_ and FF values. However, the PCE improvements of P3BTs after annealing seem much more pronounced than those of P3HTs with comparable MWs. Except for that of P3OT-52, the MW-dependent PCEs of the P3OTs remained fairly unaffected by the annealing. Actually, the MW dependencies of FF and J_SC_ of the P3OTs were found to be exactly opposite to each other, which lead to an invariance in the function of MW after multiplication.

**Figure 11:**
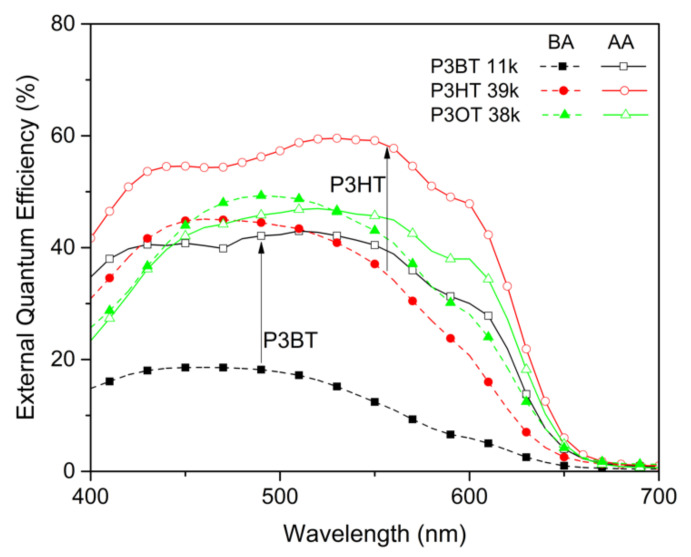
EQE spectra of selected P3ATs before (solid symbols) and after (open symbols) annealing. (BA = Before annealing, AA = After annealing).

An interesting trend was observed in the case of the PCEs of the P3ATs with shorter MWs: P3BTs with short side-chains exhibited the most pronounced PCE and significant J_SC_ improvements after annealing, while the P3HTs with moderately long side-chains showed an irregular PCE trend with only a moderate increase in J_SC_. P3OTs with long side-chains showed virtually the same PCEs, but their J_SC_ decreased upon annealing. Thus, the aforementioned observations indicate that the relationship between the side-chain lengths of the polymers and the device performance is complex; however, J_SC_ trends with relatively lower MWs might be explained in conjunction with the DSC results as follows: the DSC analysis revealed that P3ATs with short side chains exhibit lower crystallinities than those of P3ATs with longer side chains. Therefore, it can be expected that the acceptor blended solid states of P3BT may be more homogenously dispersed than those of the P3HTs and the P3OTs, which can prevent the formation of well-defined charge carrier-generating interfaces and transport channels for charges according to their polarities by the means of proper phase separation. This consideration regarding the J_SC_ difference can be compared with the difference in the EQE spectra shown in Figure 11 because EQE spectrum is spectrally resolved J_SC_ which can be regarded as a measure of the photogenerated and extracted charge carriers [65]. Nevertheless, the differences caused by the length of the side-chains disappeared dramatically upon annealing, which caused the preferable phase separation within the bulk of the active layer. However, the P3OT with short side-chains showed the opposite trend, in that their J_SC_ was significantly reduced upon annealing. These results suggest that the higher solubility and excess energy provided by annealing can be a driving force for a high degree of crystallinity. This high degree of crystallinity can also be a drawback because the crystalline domain size might be too large and may hinder the creation of effective exciton separating interfaces. However, this trend is again similar to that of other P3ATs when the MW of the P3OT increased. This observation can be explained by the additional role of the long chains in annealing. It is likely that the longer chains of P3OT hinder the formation of the oversized grains with conformational restrictions and/or entanglements, which can limit the movement of polymers in their bulk states. To connect the behaviors of the polymers in bulk states with their ensemble characteristics, each point must be treated separately as a unique scenario.

Figure 11 shows the EQE spectra of relevant P3ATs with optimal PCEs for polymers with the same side-chain lengths. Notably, P3ATs with other MWs exhibit similar spectral distributions. This indicates that the EQE spectra can be scaled with their amplitudes if a P3AT series has the same side-chain group.

However, the spectral shapes change upon annealing because the pronounced interchain interaction-induced bands centered at 602 nm coincide with the UV–Vis absorption spectra. The EQE amplitudes of P3BT and P3HT increased after the annealing treatment, whereas that of P3OT did not increase effectively. The spectral range of the band at 602 nm of the P3OTs increased; however, the range between 450 nm and 520 nm decreased correspondingly. Here, the OPV devices can capture more incident energy under the same condition. Therefore, the PCEs of most P3OTs were slightly reduced, except for those of the longest MW with P3OT-72. In addition, the spectral shapes of P3BT and P3HT changed after annealing by enhancing the interchain interaction-induced bands near 602 nm; however, the increase in their overall amplitudes was much higher than those of the P3OTs.

## 4. Conclusions

P3ATs with various side-chain groups, including butyl, hexyl, and octyl groups, were successfully synthesized using Grignard metathesis. The fabricated P3ATs exhibited well-defined MWs, narrow PDIs, and high RRs. The solubilities of the synthesized P3ATs were estimated in various solvents, and it was found that the P3OTs have relatively high solubilities in conventional organic solvents. GPC results in conjunction with the solubility information reveal that the typical overestimation of such experimental MWs compared to theoretically predicted MWs can be effectively reduced in the case of P3OTs because of their relatively high solubility in THF. T_m_ and T_C_ values of P3ATs obtained by DSC show that the crystallinity of their solids depends significantly on the MWs and the side-chain lengths, as P3OTs with higher MWs must have the highest crystallinity. The UV–Vis absorption spectra of the solutions showed a clear dependence on MW, which indicates the extended ECL of the conjugated polymers. Similar MW dependencies were observed with the PLQYs and UV–Vis spectra of the pristine and blended films. The UV–Vis spectra of the solid samples exhibited a pronounced red shift with interchain interaction-induced bands at 602 nm. Interestingly, pristine films of various P3ATs showed a clear side-chain length dependency as the relative heights of the 602 nm bands increased with increasing side-chain lengths; however, the pronounced deviation weakened upon annealing. Finally, a series of P3ATs were prepared as bulk heterojunction OPV devices, and their performance was investigated by the means of J-V characteristics and EQEs. The optimal PCE values after annealing were observed for P3BT-11, P3OT-38, and P3HT-39. The obtained device parameters were analyzed with the thermal and opto-electrical properties of the P3ATs to explore their MW and side-chain length dependencies.

## Figures and Tables

**Table 1:** Control of P3AT MW *.

P3AT	[1]_0_/[Ni]_0_	Yield (%)	Mn (kDa) ^a^	n(Thiophene Units) ^b^	Mw (kDa) ^a^	PDI ^a^	Mn (kDa) ^c^	RR (%) ^c^
P3BT-11	110	-	11.3	82	15.7	1.39	-	98
P3BT-17	110	-	17.4	126	24.0	1.38	-	98
P3BT-23	110	-	23.1	167	353	1.53	-	98
P3HT-15	71	85.7	15.2	91	20.8	1.37	12.0	98
P3HT-21	83	86.6	21.1	127	29.9	1.42	14.0	98
P3HT-39	120	81.0	39.2	236	56.0	1.43	20.0	98
P3HT-52	154	82.2	52.3	315	68.5	1.31	-	99
P3HT-61	200	67.2	61.1	368	91.7	1.50	-	98
P3HT-70	250	68.8	70.8	427	99.1	1.40	-	98
P3HT-72	333	57.9	72.3	435	108.5	1.50	-	99
P3HT-72a	500	53.3	72.4	436	108.6	1.50	-	99
P3HT-72b	1000	24.4	72.6	436	103.1	1.42	-	98
P3OT-12	67	54.0	12.2	63	16.3	1.34	13.6	98
P3OT-13	83	63.6	13.7	71	17.3	1.26	16.6	97
P3OT-18	110	66.4	18.8	97	25.2	1.34	21.5	99
P3OT-38	167	70.8	38.1	196	51.4	1.35	32.4	98
P3OT-72	333	73.1	72.2	372	111.9	1.55	-	98

* Time of Grignard-exchanged reaction: 2 h, polymerization time: 48 h. ^a^: estimated by GPC; ^b^: numbers of thiophene units; ^c^: estimated by ^1^H-NMR spectroscopy.

## Supplementary Materials

The following are available online at https://www.mdpi.com/article/10.3390/polym13193440/s1, Scheme S1: Proposed living chain growth mechanism for the synthesis of rr-P3AT by GRIM polymerization. Figure S1: Conversion of 2-bromo-3-hexyl-5-iodothiophene (1b) versus Grignard-exchanged reaction time. Figure S2: NMR spectra of 1a quenched with water at different time intervals. Figure S3: 1H-NMR used to calculate Mn of P3HTs and P3OTs. Figure S4: NMR spectra of P3HTs and P3OTs for calculated RR. Figure S5: Normalized GPC curves of P3Ats. Figure S6: UV–Vis magnification of P3Ats. Figure S7. J-V characteristics of all P3ATs before annealing (solid symbols) and after annealing (open symbols). Table S1: Solubility of P3AT in common organic solvents. Table S2: Thermal properties and crystallinity of P3ATs. Table S3: Overview of P3ATs’ device parameters.
