# Supplementary material for: Molecular Weight-Dependent Physical and Photovoltaic Properties of Poly(3-alkylthiophene)s with Butyl, Hexyl, and Octyl Side-Chains
Source: Polymers (Basel). 2021 Oct 7;13(19):3440. doi: 10.3390/polym13193440 (PMC8512356; doi:10.3390/polym13193440)
Supplement: Supplementary file 1 [file polymers-13-03440-s001.zip › polymers-1383017-SM.pdf]

Supporting Information

Molecular Weight-Dependent Physical and Photovoltaic Properties of Poly(3-alkylthiophene)s with Butyl, Hexyl, and Octyl Side-Chains

Thanh-Danh Nguyen[1,2], Van Hai Nguyen[1], Jongwoo Song[1], Jongdeok An[1], Ngoc-Thuan Truong[1], Chi-Hien Dang[2], and Chan Im[1,*]

[1]Dept. of Chemistry, Konkuk University, 120 Neungdong-ro, Gwangjin-gu, Seoul 05029, Korea

[2]Institute of Chemical Technology, Vietnam Academy of Science and Technology, 1A TL29 street, Thanh Loc Ward, District 5, Ho Chi Minh City, Vietnam.

*Email: chanim@konkuk.ac.kr

Fax: +82-2-2201-0407

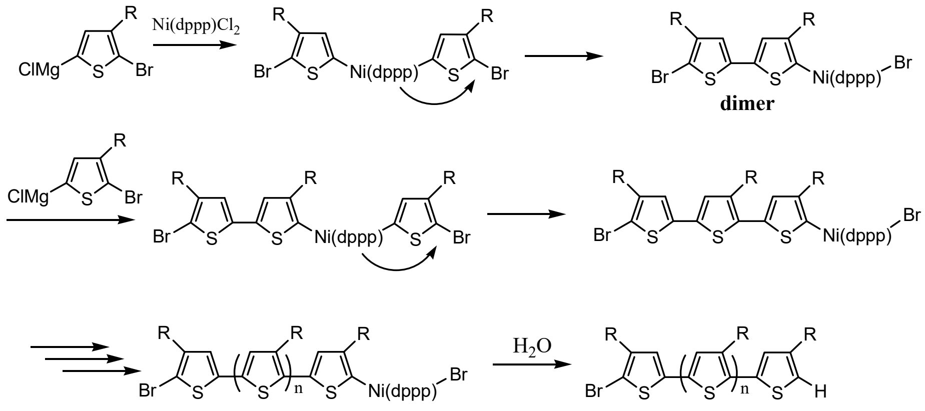

Scheme S1. Proposed living chain growth mechanism for the synthesis of rr-P3ATs by GRIM polymerization.

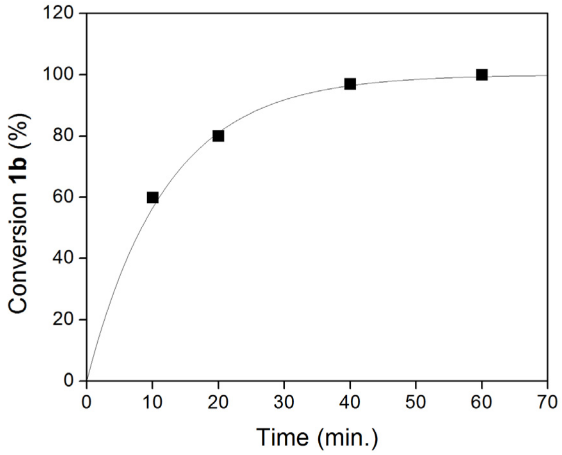

Figure S1. Conversion of 2-Bromo-3-hexyl-5-iodothiophene (**1b**) versus Grignard-exchanged reaction time.

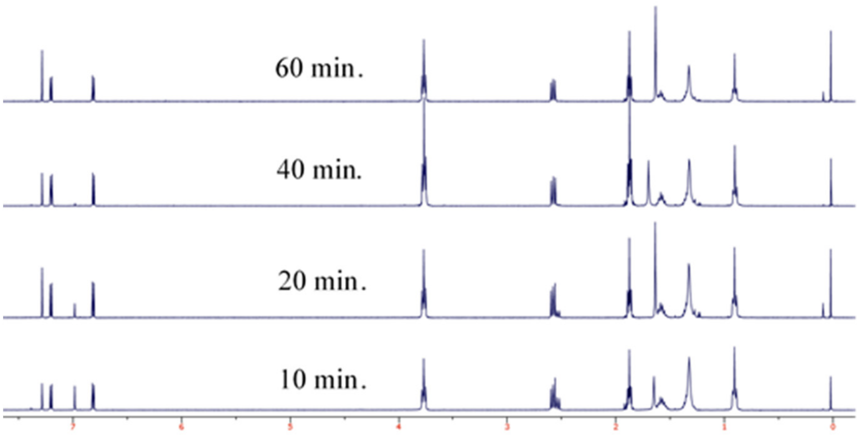

Figure S2. NMR spectra of 2-Bromo-3-hexyl-5-iodothiophene (**1b**) quenching with water at different time intervals.

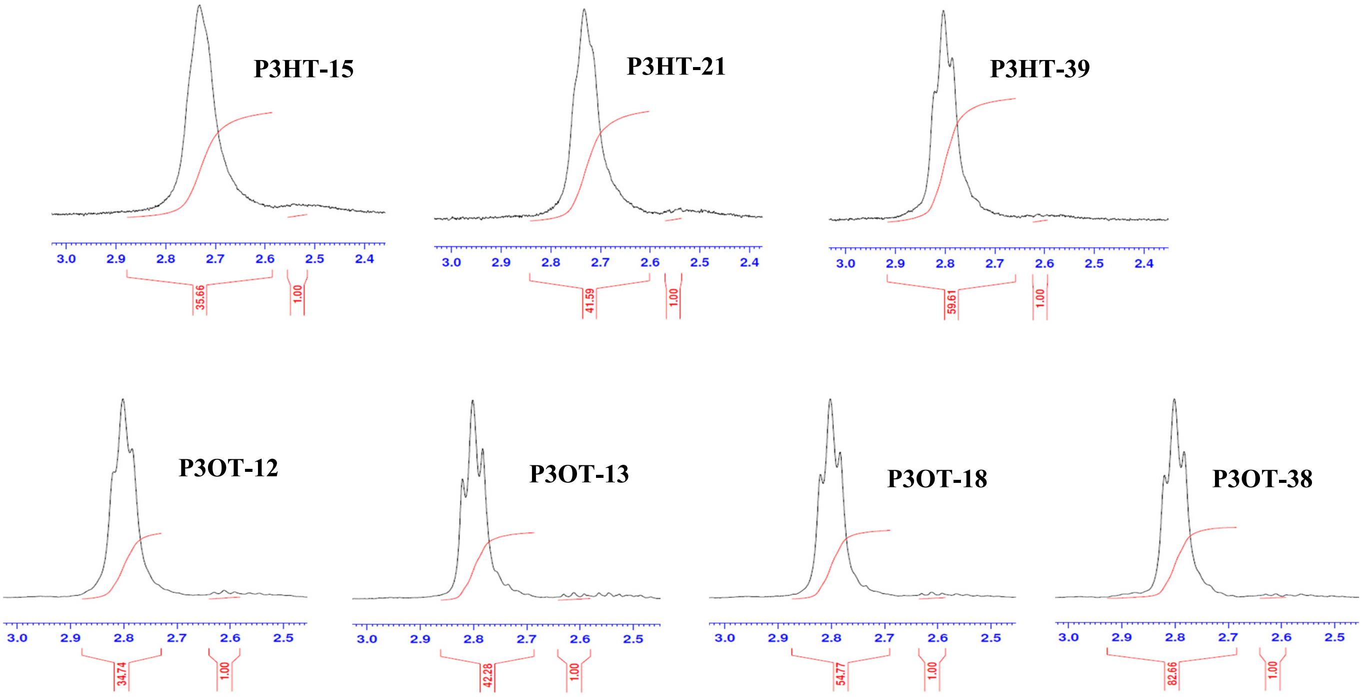

Figure S3. ^{1}H-NMR used to calculate M_n of P3HTs and P3OTs.

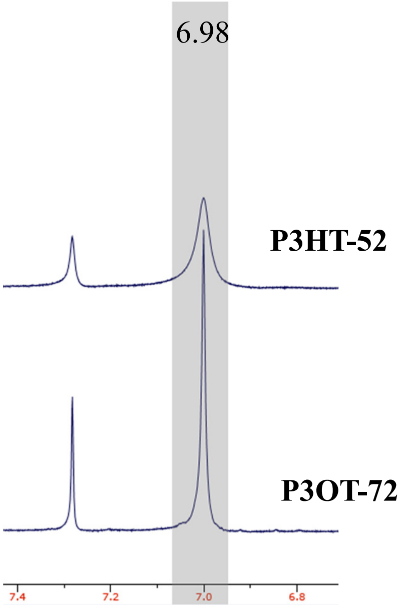

Figure S4. NMR spectra of P3HT and P3OT for calculated RR.

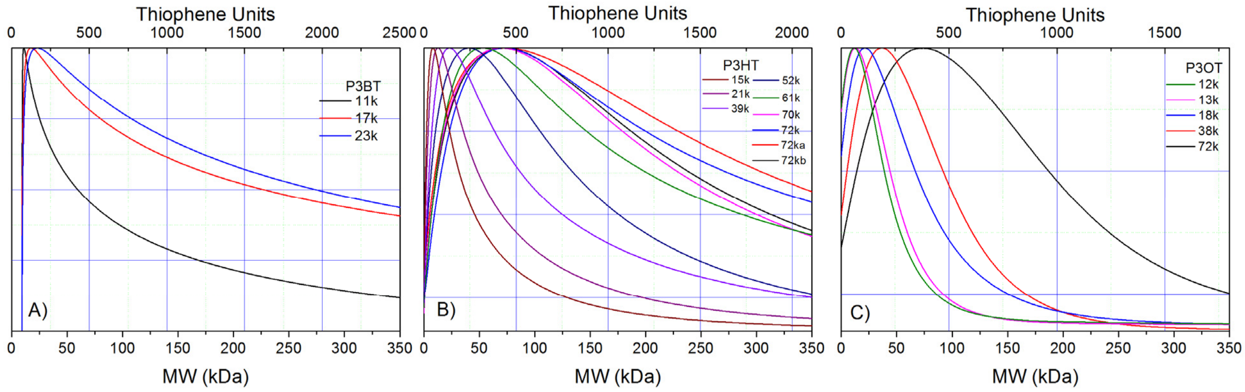

Figure S5. Normalized GPC curves of P3ATs.

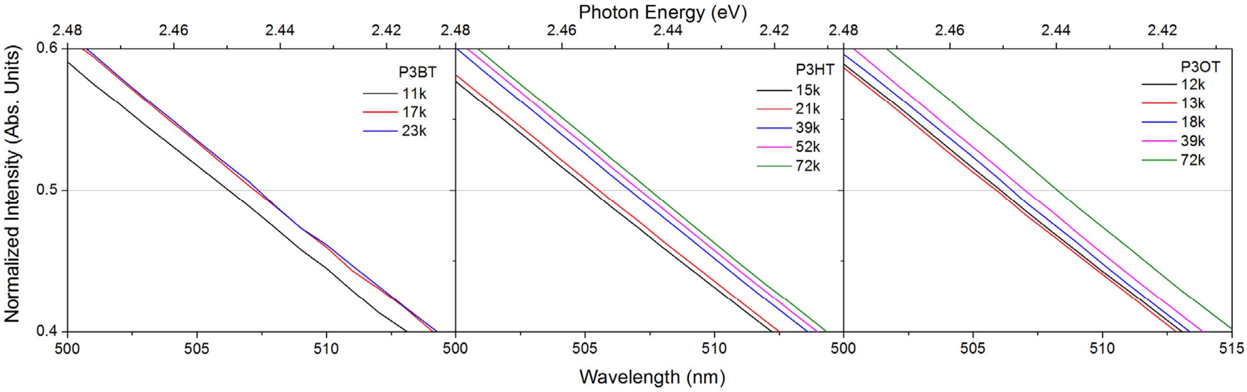

Figure S6. UV-Vis magnification of P3ATs.

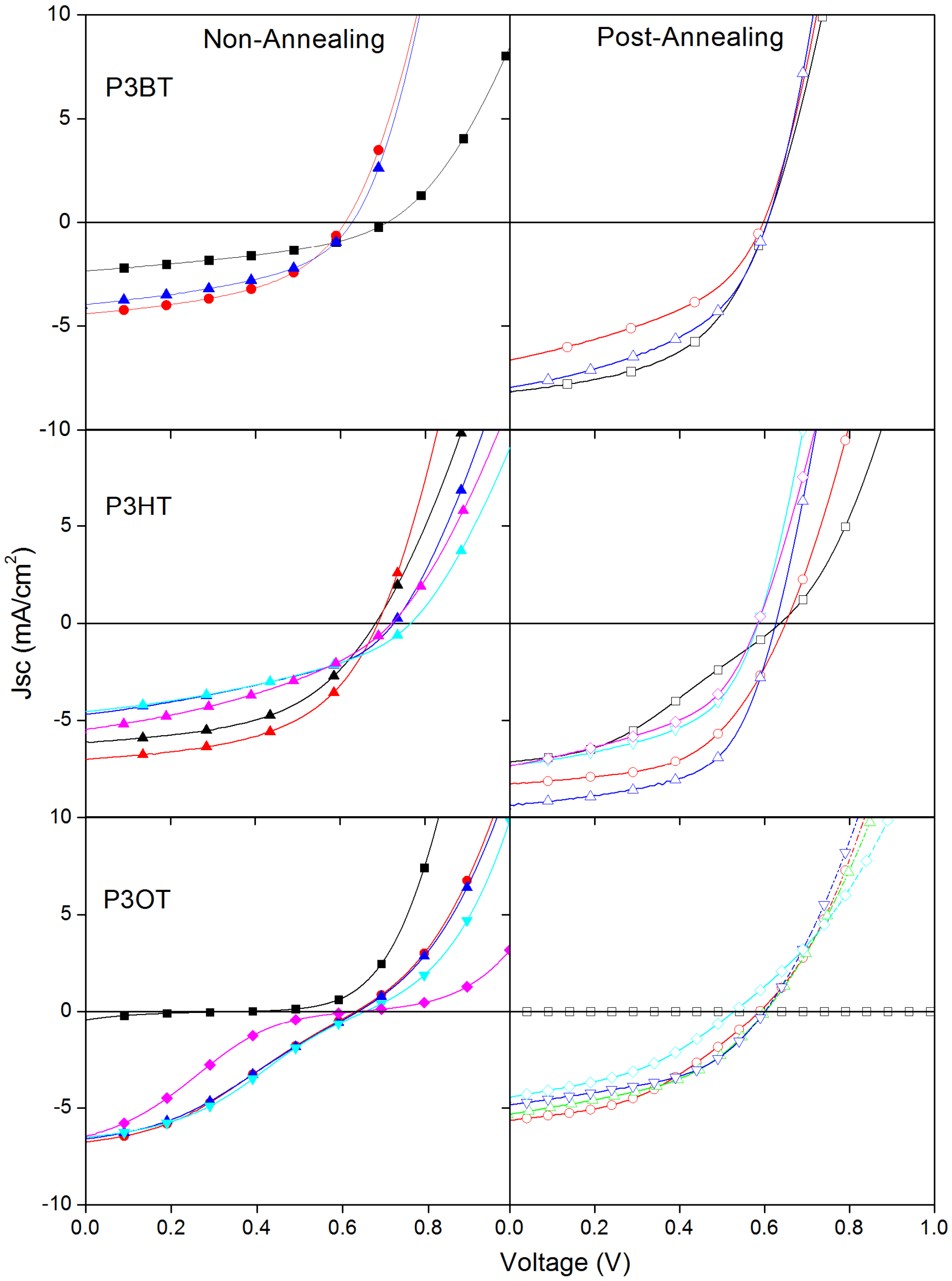

Figure S7. J-V characteristics of all P3ATs before annealing (solid symbols) and after annealing (open symbols).

Table S1. Solubility of P3ATs in common organic solvents.

P3AT	Hexane	CH_2Cl_2	THF	$CHCl_3$	Toluene	CB	ODCB
P3HT-15	MS[a]	MS[a]	S	S	S	S	S
P3HT-21	I	MS[a]	S	S	S	S	S
P3HT-39	I	MS[a]	S[a]	S[a]	S	S	S
P3HT-52	I	I	S[a]	S[a]	S	S	S
P3HT-61	I	I	S[a]	S[a]	S[a]	S[a]	S[a]
P3HT-70	I	I	S[a]	S[a]	S[a]	S[a]	S[a]
P3HT-72a	I	I	S[a]	S[a]	S[a]	S[a]	S[a]
P3OT-12	MS[a]	S[a]	S	S	S	S	S
P3OT-13	MS[a]	S[a]	S	S	S	S	S
P3OT-18	MS[a]	MS[a]	S	S	S	S	S
P3OT-38	I	MS[a]	S	S	S	S	S
P3OT-72	I	MS[a]	S	S	S	S	S

S: soluble, I: insoluble, MS: marginally soluble, a: only possible in hot solvents.

Table S2. Thermal properties and crystallinity of P3ATs.

P3ATs	T_m (ºC)	T_c (ºC)	ΔH_f (J/g)	X (%)
P3BT-11	245	165	21.7	-
P3BT-17	250	170	19.8	-
P3BT-23	249	168	25.2	-
P3HT-15	212	174	3.2	3.2
P3HT-21	220	159	4.2	4.2
P3HT-39	219	182	8.6	8.7
P3HT-52	225	189	16.4	16.6
P3HT-72a	225	180	12.1	12.2
P3OT-12	173, 184	145	11.2	14.5
P3OT-13	183, 189	155	17.5	22.7
P3OT-18	186, 191	162	14.1	18.3
P3OT-38	194	165	17.6	22.9
P3OT-72	198	162	19.7	25.6

Table S3. Overview of P3ATs' device parameters.

P3ATs	V_{oc} (V)		J_{sc} (mA/cm^2)		FF (%)		PCE (%)	
	NA	PA	NA	PA	NA	PA	NA	PA
P3BT-11	0.70	0.60	2.6	8.2	41.6	51.3	0.8	2.4
P3BT-17	0.62	0.60	4.9	7.8	47.3	42.8	1.5	2.0
P3BT-23	0.63	0.61	4.4	7.7	46.4	45.4	1.3	2.0
P3HT-15	0.69	0.65	7.1	7.9	53.0	40.0	2.4	2.0
P3HT-21	0.69	0.65	7.1	8.9	51.8	56.1	2.6	3.3
P3HT-39	0.74	0.63	5.0	9.5	44.0	61.3	1.4	3.6
P3HT-52	0.77	0.61	5.3	9.7	40.9	56.4	1.5	3.4
P3HT-72a	0.72	0.60	5.2	8.2	38.0	47.0	1.6	2.3
P3OT-13	0.63	0.57	7.0	5.5	32.4	41.3	1.4	1.4
P3OT-18	0.63	0.58	6.7	4.7	33.7	40.5	1.4	1.3
P3OT-38	0.65	0.60	6.7	5.3	35.9	46.6	1.6	1.5
P3OT-52	0.53	0.64	4.5	6.5	39.5	22.8	0.9	0.9

NA: non-annealed, PA: post-annealed.
